# Facebook Platform as the Social Business and Marketing Model for Cosmetic Surgery: An Online Study

**Published:** 2014-01

**Authors:** Muhammad Javed, Nick Wilson-Jones

**Affiliations:** The Welsh Centre for Burns and Plastic Surgery, Morriston Hospital, Swansea, United Kingdom

**Dear Editor**

Facebook ^(TM)^ with 1.11 billion active monthly users worldwide has successfully proved its worth as a business platform for game developers.^1^^,^^2^ In United Kingdom alone, around 34 million hours are spent on Facebook each day.^3^ With the wide global use of Facebook ^(TM)^, it offers an innovative and invaluable platform for marketing small and large businesses. The use of social media in plastic surgery practice is not new and in one recent study, 52.1% plastic surgeons believed it to be an effective marketing tool and 33.8 % reported a positive impact of social media on their practice.^4^^-^^6^

In this online study, we evaluated the existing extent of cosmetic surgery marketing on Facebook ^(TM)^ and the ease of setting up a business page for social marketing.

An online search was carried out on Facebook^(TM)^ search tab using search terms ‘Cosmetic Surgery’, ‘Plastic Surgery’ and ‘Botox’. Only pages intended for Facebook ^(TM)^ business were included in the study. Secondly, an experimental page was created using the Facebook’s ^(TM)^ ‘Create a page’ option. Online search revealed a total of 1059 business pages. 48.2% (n=511) pages advertised plastic surgery. 18.31% (n=194) were dedicated to Botox and 33.4% (n=354) to cosmetic surgery. The Facebook ^(TM)^ interfaced for developing business pages. We created a mock cosmetic surgery page for advertising. The whole process took less then 5 minutes to setup a basic and acceptable page. The interface which can be customised according to developers need for marketing further offers advertiser and customer or fan interactions by discussion threads, comments, running contests, creating articles, press releases, and links for advertiser’s own web site content. The facility to create such pages is free of charge through the self service interface however social business plan for plastic surgeons can also benefit from the use of Facebook’s ^(TM)^ commercial advertising. 

The use of social media in plastic surgery practice is likely to grow and Facebook ^(TM)^ can potentially provide a very cheap and interactive consumer based cosmetic surgery marketing platform; however, concerns regarding appropriate and ethical cosmetic surgery advertising need to be addressed.

## CONFLICT OF INTEREST

The authors declare no conflict of interest.

## Footenote

Please cite this paper as:

Javed M, Wilson-Jones N. Facebook Platform as the Social Business and Marketing Model for Cosmetic Surgery: An Online Study. World J Plast Surg 2014;3(1):68-69.
